# Genomic consequences of transitions from cross- to self-fertilization on the efficacy of selection in three independently derived selfing plants

**DOI:** 10.1186/1471-2164-13-611

**Published:** 2012-11-12

**Authors:** Rob W Ness, Mathieu Siol, Spencer C H Barrett

**Affiliations:** 1 Department of Ecology and Evolutionary Biology, University of Toronto, Toronto, ON, M5S 3B2, Canada; 2 Centre for the Analysis of Genome Evolution and Function, University of Toronto, Toronto, ON, M5S 3B2, Canada; 3 UMR Agroécologie, INRA, BP86510, 17 Rue Sully, Dijon, 21000, France

## Abstract

**Background:**

Transitions from cross- to self-fertilization are associated with increased genetic drift rendering weakly selected mutations effectively neutral. The effect of drift is predicted to reduce selective constraints on amino acid sequences of proteins and relax biased codon usage. We investigated patterns of nucleotide variation to assess the effect of inbreeding on the accumulation of deleterious mutations in three independently evolved selfing plants. Using high-throughput sequencing, we assembled the floral transcriptomes of four individuals of *Eichhornia* (Pontederiaceae); these included one outcrosser and two independently derived selfers of *E*. *paniculata,* and *E*. *paradoxa,* a selfing outgroup. The dataset included ~8000 loci totalling ~3.5 Mb of coding DNA.

**Results:**

Tests of selection were consistent with purifying selection constraining evolution of the transcriptome. However, we found an elevation in the proportion of non-synonymous sites that were potentially deleterious in the *E*. *paniculata* selfers relative to the outcrosser. Measurements of codon usage in high versus low expression genes demonstrated reduced bias in both *E. paniculata* selfers.

**Conclusions:**

Our findings are consistent with a small reduction in the efficacy of selection on protein sequences associated with transitions to selfing, and reduced selection in selfers on synonymous changes that influence codon usage.

## Background

Transitions from cross-fertilization to self-fertilization can have profound effects on population genetic structure and patterns of molecular evolution across the genome [1]. Most importantly, homozygosity increases with more intense selfing, which decreases effective population size (*N*_*e*_) and reduces opportunities for crossing over between heterozygous sites, resulting in elevated linkage disequilibrium [2,3]. Effective population size is also reduced around selected regions by the effects of genetic hitchhiking, including selective sweeps of beneficial mutations and background selection against deleterious mutations (reviewed in [4]). Linkage among weakly selected sites with opposing selective forces can also interfere with the ability of selection to act efficiently [5].

Estimates of *N*_*e*_ in selfers are often lower than the expected two-fold decrease based on selfing alone. This result presumably occurs because life-history characteristics associated with selfing promote population subdivision, isolation, and frequent genetic bottlenecks [6-10]. Thus, both genetic and demographic processes in selfing populations should lead to a decrease in the efficacy of natural selection and an increase in the fixation of slightly deleterious mutations, with important consequences for genome evolution. The accumulation of deleterious mutations may also be an important factor in causing species extinction [11], and could explain the lack of persistence of selfing lineages over longer time scales [12-14]. However, the extent to which theoretical predictions on the reduced efficacy of selection in selfing populations occur is unclear.

The efficacy of selection depends on the product of *N*_*e*_ and the selection coefficient (*s*). The reduction of *N*_*e*_ due to selfing is expected to result in a higher rate of fixation of slightly deleterious mutations and a lower rate of fixation for advantageous mutations (reviewed in [4]). Diverse methods have been developed to detect the footprint of natural selection at the molecular level (reviewed in [15,16]). One common approach is to quantify the ratio of mutations at non-synonymous sites (*d*_*N*_) versus synonymous or silent sites (*d*_S_); hereafter ω. Because selection acts primarily on proteins and not DNA sequences, synonymous changes are often treated as selectively neutral (but see below) thus enabling measurement of the degree of selective constraint on amino acid sequences. Under neutrality ω is expected to equal 1, whereas values of ω less than or greater than 1 indicate purifying or positive selection, respectively. The vast majority of functional proteins that have been examined exhibit ω values much less than one indicating that most protein sequences are subject to purifying selection. However, in selfers a reduction in the efficacy of selection may result in elevation of this value as a result of the accumulation of deleterious mutations.

Although predictions concerning the effect of selfing on levels of polymorphism have been well documented [17,18], evidence for a reduction of selection efficacy in selfing populations of diverse plants and animals is equivocal. The only study that we are aware of that has attributed an accumulation of deleterious mutations to selfing was a comparison of the selfing plant *Arabidopsis thaliana* with *Drosophila melanogaster*[19]. Other studies focusing on closely related outcrossers and selfers [20-25] have failed to detect convincing evidence of reduced selection efficacy leading to several hypotheses to explain the apparent lack of signal in the molecular data. First, the genomic distribution of selection coefficients is poorly known [26,27], and if there are few weakly selected mutations very little effect of the mating system is predicted [28]. Another explanation involves the amount of time that has elapsed since the transition from outcrossing to selfing, which in some instances may be too short for substantial changes to have occurred at the genome level [1]. Finally, no comparisons have been made using genomic data involving very large numbers of loci (*e.g.* thousands of genes) and it is possible that larger genomic samples may allow the detection of weak effects of selfing despite the stochasticity and slow rate of mutation accumulation.

The reduced efficacy of selection due to decreased *N*_e_ is also predicted to diminish the signal of biased codon usage. Proportional usage of synonymous codons often differs between high and low expression genes due to selection for higher translational efficiency and accuracy [29,30]. However, the strength of selection on synonymous codon usage is expected to be weak relative to purifying selection against amino acid altering mutations. If this is true, reductions in *N*_*e*_, such as those associated with transitions to selfing, are predicted to reduce the efficacy of selection to the extent that these mutations become effectively neutral [31,32]. Thus, in principle the reduced efficacy of selection can be inferred from an increase in the frequency of substitutions (or polymorphisms) for unpreferred codons, or by less differentiation of codon usage between high and low expression genes. However, because codon bias is eroded by genetic drift this process will occur rather slowly and is dependent on the rate of mutation [33]. Therefore, to detect the reduced efficacy of selection based on codon usage it may be necessary to include either old selfing lineages, which may be difficult given the relatively recent origin of most selfers [14,34], or to obtain data from a very large number of loci, to detect the small changes that are possible in codon usage, an approach we use here.

Empirical tests of these predictions have included selfing and outcrossing species of Brassicaceae [22,35,36], *Caenorhabditis*[23,37] and members of the Triticeae [24,25]. The results of these studies are mixed, with no evidence of reduced codon bias in selfing species of the Triticeae, a slight reduction in codon bias in selfing *C*. *briggsae* relative to outcrossing *C*. sp. 5, and a recent analysis found evidence of an effect of selfing on codon usage in Brassicaceae [36]. Two explanations have been proposed for the lack of a strong effect of selfing on codon usage. First, as mentioned above, many selfing lineages are thought to be of recent origin (*e.g.*[37]), and there simply may not have been sufficient time for enough mutations to have drifted to fixation. Second, interspecific comparisons introduce confounding effects that are not directly related to selfing because of the independent evolutionary history of the species, thus limiting conclusions about the effect of selfing on codon bias [22]. Ideally, predictions require contrasts between conspecific selfing and outcrossing lineages with fewer confounding effects; however, this may not be feasible over long evolutionary time spans. The approach we use in this study is to contrast both inter and intraspecific selfing lineages of different ages and to use a large sample of loci in an effort to detect changes in the efficacy of selection on genomes.

Here, we investigate patterns of molecular evolution in the floral transcriptomes of three independently derived selfing lineages relative to an outcrossing genotype in *Eichhornia* (Pontederiaceae), a neotropical genus of aquatic plants. Our samples include three individuals of *E. paniculata*, an annual diploid that has been the subject of detailed studies on the ecology and genetics of mating-system variation over several decades (reviewed in [38]). Populations of *E. paniculata* are largely concentrated in N.E. Brazil, with smaller foci in Jamaica and Cuba and isolated localities in Nicaragua and Mexico. Populations in Brazil are mostly outcrossing and possess the sexual polymorphism tristyly, which promotes cross-pollination among the three floral morphs (reviewed in [39]). Morphological, genetic and biogeographical evidence indicates that tristyly has broken down on multiple occasions in *E. paniculata* resulting in independently derived selfing populations [38,40,41]. Populations from Jamaica are largely composed of selfing variants of the mid-styled morph (M-morph) in which stamens are elongated to a position adjacent to mid-level stigmas resulting in the autonomous self-fertilization of flowers. In contrast, plants from Mexico and Nicaragua are selfing variants of the long-styled morph (L-morph) with a different arrangement of sexual organs (see Figure two in [38]). Although both variants possess the selfing syndrome, comparisons of molecular variation at 10 EST-derived nuclear loci indicate a high level of genetic differentiation consistent with their separate origins from different outcrossing ancestors (see Figure three in [38]). Our analysis included an individual of both selfing variants and an individual of an outcrossing L-morph from N.E. Brazil, the likely centre of origin of the species. We also included a selfing individual of *E. paradoxa*, the sister species of *E. paniculata* (*e.g.*[42]), to serve as a potentially more ancient selfing phenotype, and as an outgroup for inferences on the ancestral DNA sequence in our samples. Most populations of *E. paradoxa* are predominantly selfing, although a tristylous population is known from Brazil (see [43]) indicating that, in common with *E. paniculata*, selfing has likely arisen from the evolutionary breakdown of tristyly.

We used high-throughput DNA sequencing technology to generate a set of approximately 8000 orthologous ESTs from the floral transcriptomes of the four *Eichhornia* genotypes. Using this dataset we investigated the molecular evolution of protein coding genes to address the following specific questions predicted by the hypothesis of reduced selection efficacy in selfers: (1) Is there evidence for relaxation of purifying selection against non-synonymous mutations in selfing lineages? (2) Can we detect biased codon usage in our samples, and if so, does this vary among lineages based on their mating systems?

## Methods

### Samples, tissue preparation and sequencing

We selected the four plants used in our study from glasshouse collections maintained at the University of Toronto. These were originally obtained by germinating open-pollinated seed collected from field populations at the following localities: outcrossing L-morph B211, Fortaleza, Ceará, N.E. Brazil; selfing M-morph J16, Georges Plain, Westmoreland, Jamaica; selfing L-morph N1, Rio Las Lajas, Rivas, Nicaragua; *E. paradoxa,* Patos, Paraíba, N.E. Brazil.

We extracted total RNA from floral tissue sampled evenly throughout development from bud to flower. We used the Illumina (San Diego, CA) mRNA-Seq, paired-end protocol on a Genome Analyzer, GAII, for 40 cycles. This resulted in an average of ~1.55 × 10^9^ bp of sequence per sample. We performed *de novo* assembly on each sample separately using the program Oases (D. R. Zerbino, European Bioinformatics Institute). We then generated a consensus transcriptome by identifying orthologous sequences among the four samples using a reciprocal best BLAST approach. The consensus sequence of these orthologs was used as a reference on which we aligned the original reads. This approach allowed us to use the frequency of bases at each position to statistically infer each genotype. To call the genotypes we used the method implemented in the software Maq and SamTools [44,45] with a quality threshold of *Q* > 20 (*P* = 0.01), where *Q* is the Phred-scaled probability that the consensus genotype call is wrong. Sites for which we could not determine the genotype, with at least this level of confidence, were marked as ambiguous. To detect potential errors in read mapping we assumed that selfing genotypes were largely homozygous, and therefore the presence of the same heterozygous sites in multiple selfing genotypes would indicate errors in read mapping, possibly due to paralogous sequences. We excluded these loci from downstream analyses. Read mapping also allowed us to use the abundance of reads derived from each locus to estimate gene expression. We calculated the number of fragments per kilobase per million fragments mapped (FPKM) with the program Cuffdiff from Cufflinks v 0.83 [46]. Full details on the sequencing, assembly, quality assessment and expression analysis can be found in Ness et al. [47].

### EST analysis, filtering and alignments

To assign open reading frames and repair potential indels inserted during transcript assembly we used the pipeline prot4EST [48]. We started from an extended list of 10,263 single-copy ESTs that were assembled in all four genotypes. Nucleotide sequences were aligned on the basis of their translated amino-acid sequences with the software Muscle [49] using the backalign function of the EggLib package [50]. We removed alignments with frame shifts and the remaining alignments were confirmed visually. Our final dataset was comprised of 7890 alignments totalling 4,952,907 bp of sequence and these were used for all subsequent analyses. By comparing Illumina and Sanger data over 7977 bp of aligned sequence we estimated a conservative error rate of 3.76 × 10^-4^ errors/site (see Additional file 1 for details of our SNP validation method). This error rate is two orders of magnitude lower than the estimate of diversity in our samples, and not likely to influence the inferences presented here.

### Selection on protein coding changes

We investigated patterns of selection on coding sequence as changes in the proportion of non-synonymous and synonymous derived mutations in each *E. paniculata* genotype, using the outgroup *E*. *paradoxa* to infer the ancestral state by parsimony. Misinference of the ancestral state through violations of infinite sites or ancestral polymorphism was unlikely to affect our analyses because we used the ratio of *d*_*N*_ /*d*_*S*_ to compare among lineages. Moreover, there is no reason to expect more or less multiple mutations or ancestral polymorphisms in the selfing or outcrossing lineages and any noise introduced to the analysis should be unbiased. Using simple counts relies on few underlying assumptions and alleviates the need for divergence data between lineages, which is required by the *d*_*N*_ /*d*_*S*_ approach described in the introduction. We then tested for significant differences in the proportion of derived non-synonymous to synonymous mutations in each individual using a *χ*^2^ homogeneity test with two degrees of freedom. Heterozygous sites were taken into account by generating two unphased haploid sequences and taking the average of the counts.

To classify the potential effects of non-synonymous mutations we used the program MAPP [51]. This method relies on an alignment of translated transcripts to homologous proteins to estimate the physiochemical constraints at positions in the protein. Each position in the protein is assigned a list of compatible (‘good’) or incompatible (‘bad’) amino-acids. To test for an accumulation of deleterious mutations in the selfing lineages we conducted two analyses. First, using *E. paradoxa* to infer the ancestral state, we tested whether non-synonymous changes along each lineage were classified as deleterious. Second, to mitigate any possible problems with ancestral state assignment, we considered all polymorphic sites within *E. paniculata* and tested whether the allele from each genotype was deleterious. To generate alignments of each of our transcripts we used BLASTx to identify homologous proteins in the NCBI non-redundant (NR) protein database, keeping only the five best hits. We then conducted a global multiple-alignment using the software MUSCLE. To avoid alignment errors we retained only those with >60% identity at the protein level. We constructed a tree for each alignment using PhyML with default parameters and a BLOSUM62 substitution matrix for amino-acids [52]. The alignments and inferred trees were used as the input for MAPP.

### Assessing selection for codon bias

To measure codon usage bias we calculated the frequency of optimal codon usage (*F*_op_) for each gene from all four genotypes. Because optimal codons can vary among species we first identified these codons based on the assumption that codon bias is strongest in highly expressed genes (*e.g.*[30,53]). We therefore identified optimal codons as those which differed in their usage between high versus low expression genes using the method of Duret and Mouchiroud [30]. We first calculated the relative synonymous codon usage (RSCU) for each codon in all genes using the program codonW (J. Peden http://codonw.sourceforge.net). From this we contrasted RSCU in high expression (top 10^th^ percentile, 185,546 codons) versus low expression (bottom 10^th^ percentile, 260,695 codons) as *∆RSCU* = RSCU_high_ - RSCU_low_[30]. We calculated this measure using both the mean expression across samples and with the sample-specific expression values, but the results were the same. We measured expression as the number of sequence reads per kilobase per million reads mapped FPKM, [46]. Statistical departure of *∆RSCU* from zero was tested for each codon using a one-way analysis of variance (ANOVA) conducted in JMP v8.0 and codons with significantly positive values were inferred to be putative optimal codons. We calculated the frequency at which the optimal codon is used across sites (*F*_op_) in codonW with a customized optimal codons table. We repeated this procedure for each of the four genotypes to calculate genotype-specific tables. We also defined consensus optimal codons as those that were optimal in all four genotypes and limited this set to include only one codon per amino acid. All analyses presented here are based on the consensus optimal codons, but the results were qualitatively similar using genotype specific estimates.

Both selection and the background base composition can influence estimates of *F*_op_. We assessed the role of these factors, genotype and mating system to determine their influence on codon usage bias in our four samples. We constructed ANOVA models in JMP v8.0 where *F*_op_ was a function of base composition (measured as GC content at silent sites, GC3s), gene length (bp), gene expression (FPKM), genotype and mating system (represented as a categorical variable; selfing or outcrossing). Genotype and mating system could not be included simultaneously because there is only one outcrossing genotype (zero degrees of freedom) and they were therefore run in two separate models. Our models initially assessed the effects of all of these factors and their interactions. Terms were excluded from the model by backward elimination (*α* = 0.05) if they did not explain a significant proportion of the variation in codon bias, and they were not involved in any significant higher order interactions. Although GC3s was found to be highly significant, we also present the results of an ANOVA that excluded GC3s because we found that all of our putatively optimal codons have guanine or cytosine in the third position, therefore GC3s was not independent of *F*_op_.

The statistic ${\overset{―}{\Delta RSCU}}_{+}$ proposed by Cutter et al. [23], measures the strength of selection for codon bias as the mean of all positive values of *∆RSCU* for a given genotype. Because RSCU is independent of the amino acid content and *∆RSCU* controls for base composition, ${\overset{―}{\Delta RSCU}}_{+}$ provides a useful metric for comparing the degree of codon usage bias across genotypes and species. We calculated ${\overset{―}{\Delta RSCU}}_{+}$ for each genotype and tested for significant effects of mating system and genotype using two separate one-way ANOVAs of ${\overset{―}{\Delta RSCU}}_{+}$ among genotypes. We also compared the ${\overset{―}{\Delta RSCU}}_{+}$ values for mating system (selfing versus outcrossing) and genotype using the Tukey-Kramer HSD test. All values of ${\overset{―}{\Delta RSCU}}_{+}$ were log transformed for these statistical tests so they fitted the normal distribution.

We calculated counts of the number of synonymous mutations that resulted in changes from preferred to unpreferred codons (P2U), or unpreferred to preferred codons (U2P), using the optimal codons defined in our analysis of *∆RSCU* and the outgroup *E*. *paradoxa* to infer the ancestral state [31]. An elevation in the proportion of changes from preferred to unpreferred codons can be interpreted as a relaxation of selection for optimal codons. It should be noted, that P2U changes that may have occurred in the outgroup will potentially increase the measured rate of U2P in the branch shared by all *E*. *paniculata* genotypes; however without more outgroup sequences this cannot be quantified. We tested for significant differences among each of our *E. paniculata* genotypes and each genotype relative to the ancestral branch connecting *E. paniculata* genotypes to *E*. *paradoxa* using a *χ*^2^ homogeneity test.

## Results

Our dataset for the four *Eichhornia* genotypes consisted of 7890 loci (average length = 627.7 bp, median length = 681 bp) totalling 4,952,907 bp of aligned sequence. There were 30,119 polymorphic sites among our three samples of *E. paniculata* and 131,345 divergent sites between *E*. *paniculata* and *E. paradoxa* (see Table 1A). Mean non-synonymous polymorphism (*θ*_*WNS*_ = 0.0015) was almost an order of magnitude lower than mean synonymous polymorphism (*θ*_*WS*_ = 0.0097). There were a similar number of pairwise differences when comparing Jamaican with Brazilian (17,018.5) and Jamaican with Nicaraguan genotypes (17,201). The Brazilian and Nicaraguan genotypes had the fewest number of differences (15,485). Consistent with theory, in *E. paniculata* we found a higher frequency of heterozygous sites in the Brazilian outcrossing genotype (*H*_*obs*_ = 9.8 × 10^-4^) than in the two selfers (Jamaica *H*_*obs*_ = 1.8 × 10^-4^, Nicaragua *H*_*obs*_ = 0.8 × 10^-4^).

**Table 1:** **Summary statistics from the dataset on synonymous and non-synonymous sites across all loci of *Eichhornia***

**(A)**				
Site class	N_sites_	S	K	*θ*_W_
Synonymous	975202	19570	90113	0.0097 (0.012)
Non-synonymous	3339788	10549	41232	0.0015 (0.0022)
**(B)**				
	*E. paradoxa*	Jamaica	Nicaragua	Brazil
*E. paradoxa*	-			
Jamaica	142,005.5	-		
Nicaragua	142,579.5	17,201	-	
Brazil	142,113	17,018.5	15,485	-

(**A**) The number of aligned sites (N_sites_), the number of polymorphic sites (S), the number of divergent sites (K) and Watterson’s polymorphism estimator (*θ*_*W*_). (**B**) Number of pairwise differences between the four individuals. Note that values are the average of the pairwise comparisons of the unphased haplotypes, thus explaining why in some case the number are not integers.

### Selection on non-synonymous mutations

The *χ*^2^ test of homogeneity comparing the ratio of synonymous to non-synonymous mutations in the *E. paniculata* genotypes was significant (*P* <0.01) with a slightly higher ratio of non-synonymous to synonymous derived mutations in the selfing genotype of *E. paniculata* from Nicaragua (0.468) compared to the outcrosser from Brazil (0.438) or the selfing genotype from Jamaica (0.429). Although the magnitude of these differences is not very large, the trend suggests a slight elevation of the proportion of non-synonymous changes in the Nicaraguan selfing genotype of *E. paniculata*.

**Figure 1:**
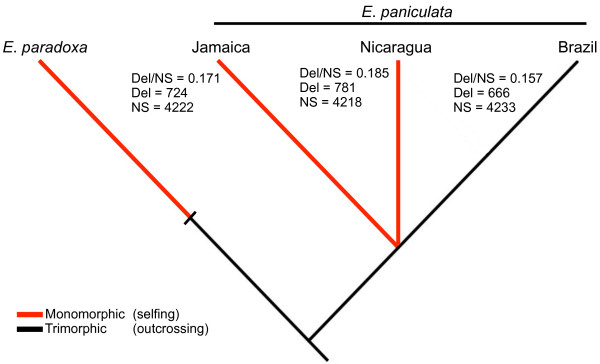
**Counts of the number of deleterious (Del) non-synonymous sites and the total number of non-synonymous (NS) sites in selfing and outcrossing genotypes of *Eichhornia paniculata.*** Deleterious mutations were identified using the program MAPP, which uses evolutionary constraint in homologous proteins to infer the potential fitness impact of non-synonymous changes. The colours along the branches reflect the putative model of changes in mating system although the exact timing of the transition along each branch is not known. The branch lengths are not to scale.

When all non-synonymous changes are categorized as deleterious or neutral, using the program MAPP, we found both selfing genotypes of *E*. *paniculata* showed a slightly higher proportion of deleterious changes (Figure 1) for the 4260 genes included in this analysis (transcripts with at least 5 homologous proteins and >60% identity in the protein alignments). When considering all non-synonymous polymorphic sites, we found a significantly higher fraction of deleterious mutations in the two selfers (Nicaragua, 781 deleterious of 4218 total sites, deleterious/total = 0.185; Jamaica, 724 deleterious of 4222 total sites, deleterious/total = 0.171) compared to the outcrosser (Brazil, 666 deleterious of 4233 total sites, deleterious/total = 0.157, *χ*^*2*^ = 8.15, *P* = 0.016). When *E*. *paradoxa* was used as an outgroup to polarize the direction of mutations the trend was similar but marginally insignificant (Nicaragua, deleterious/total = 0.268; Jamaica, deleterious/total = 0.242; Brazil, deleterious/total = 0.225, *χ*^2^ = 5.27, *P* = 0.07).

### Assessing selection for codon bias

**Figure 2:**
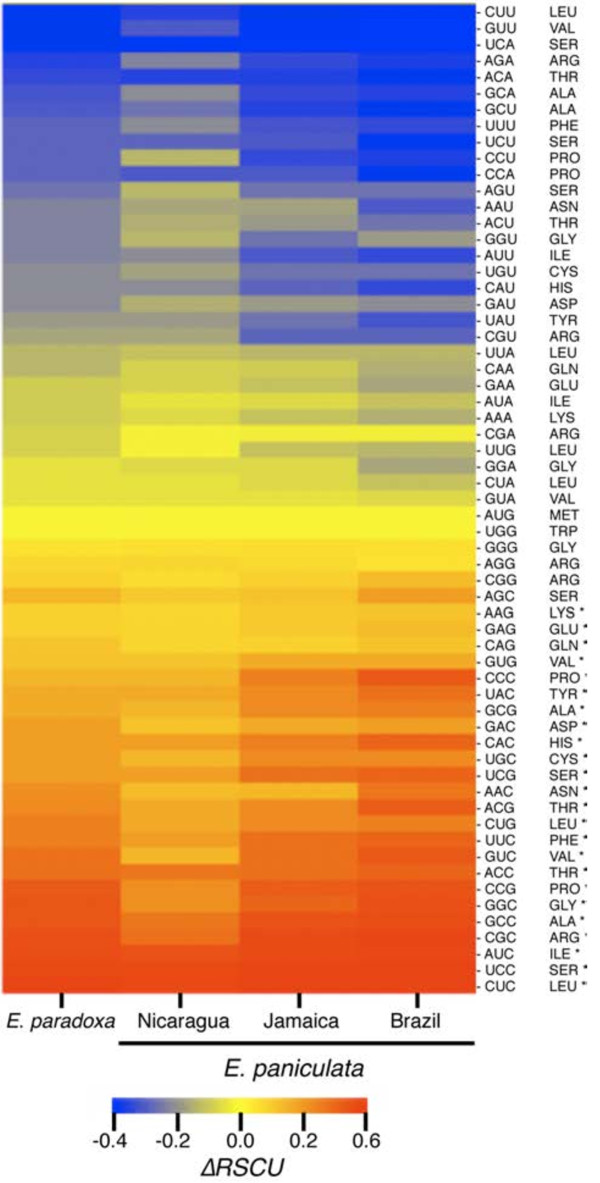
**Heat map of *ΔRSCU *values for four genotypes of *Eichhornia *included in this study.** Each column represents a single genotype. Each row corresponds to one of the 61 codons (excluding stop codons), where the value of *ΔRSCU* for each genotype is indicated. Codons are ordered by the mean value of *ΔRSCU* across all genotypes. Optimal codons identified as part of the consensus set of codons are marked with an asterisk. The figure was generated with the software CIMMiner (http://discover.nci.nih.gov/cimminer).

Codon usage in each of the four genotypes was highly non-random. Summary statistics for high and low expression genes as well as all loci combined are presented in Table 2. GC content at 4-fold degenerate sites in all genes (GC3s) was similar across all samples (GC3s_*E. paniculata*_ = 0.480, GC3s_*E. paradoxa*_ = 0.483) but was significantly different in high versus low expression genes (GC3s_high_ = 0.583, GC3s_low_ = 0.465, Tukey-Kramer HSD q* = 2.34 *P* < 0.0001). As a result, we found that values of RSCU for each codon in each genotype differed between high and low expression genes. Using *∆RSCU* analysis we identified 24 putative optimal codons (those with significantly positive *∆RSCU* values, Figure 2) that were found in all four genotypes representing 18 different amino acids. All 24 of these putatively optimal codons had guanine or cytosine in the third position. Each of the degenerate amino acids was represented by at least one putatively optimal codon. The six amino acids for which there were more than one optimal codon included alanine, leucine, proline, serine, threonine and valine. In each of these amino acids with two preferred codons, the codon with a greater *∆RSCU* value terminated with a cytosine rather than a guanine. This pattern occurred despite the higher proportion of guanine in synonymous third positions sites across all genes.

**Table 2:** **Mean values of expression, gene length, frequency of optimal codon usage (*F***_**op**_**) and base composition, measured as GC-content at synonymous sites (GC3s) for genes sampled from four genotypes of *Eichhornia***

**Expression category**	**Genotype**	**Expression (FPKM)**	**Length (bp)**	***F***_**op**_	**GC3s**
high	*E. paniculata,* Brazil	166.1	698.4	0.424	0.582
	*E. paniculata,* Jamaica	155.8	698.4	0.424	0.582
	*E. paniculata,* Nicaragua	162.2	698.4	0.424	0.581
	*E*. *paradoxa*	154.8	698.4	0.427	0.586
low	*E. paniculata,* Brazil	3.4	981.3	0.333	0.465
	*E. paniculata,* Jamaica	3.6	981.3	0.334	0.465
	*E. paniculata,* Nicaragua	3.7	981.3	0.334	0.465
	*E*. *paradoxa*	5.8	981.3	0.335	0.467
total	*E. paniculata,* Brazil	43.2	628.0	0.339	0.480
	*E. paniculata,* Jamaica	42.1	628.0	0.338	0.480
	*E. paniculata,* Nicaragua	43.2	628.0	0.338	0.480
	*E*. *paradoxa*	43.0	628.0	0.341	0.483

For each of the genotypes, the genes were divided into two categories based on expression level including the top (‘high’) and bottom (‘low’) and 10^th^ percentile of expression. The values for all loci are summarized in the category, ‘total’.

We used these 18 putatively optimal codons to calculate codon bias in each gene of each genotype. We found that GC3s had a highly significant effect on measures of codon bias (*F*_op_; *F*_6, 31813_ = 370016.2, *P* < 0.0001) explaining 88.9% of the variance in *F*_op_. In addition, both length and gene expression had significant effects on *F*_op_, but only explained 0.011% and 0.008% of the variance in *F*_op_, respectively. There was no significant effect of either mating system or genotype on the frequency of optimal codons. However, there was strong differentiation in both GC3s and *F*_op_ in high versus low expression genes, demonstrating that selection for codon bias is stronger in up-regulated genes. There was also a subtle elevation in the *F*_op_ of highly expressed genes of the outcrosser relative to all three selfers, although the trend was not significant.

**Figure 3:**
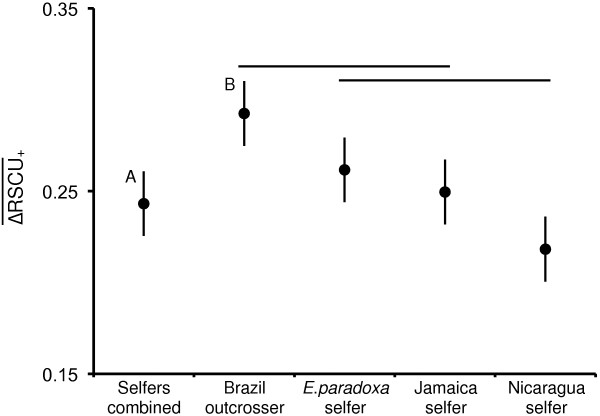
**Comparisons of codon bias, measured as**${\overset{―}{\Delta RSCU}}_{+}$**, among each of the four *Eichhornia *genotypes, and the three selfing genotypes combined.**${\overset{―}{\Delta RSCU}}_{+}$ is the mean of all positive *ΔRSCU* values for a given genotype. The results of two separate tests of significance are marked with A and B for the difference in mean ${\overset{―}{\Delta RSCU}}_{+}$ between the outcrosser and the selfers combined (*t* = 1.99, *P* < 0.01). Bars indicate significant differences among individual genotypes using a Tukey-Kramer HSD (q* = 2.62, *P* < 0.01) with error bars indicating the standard error of each estimate.

To compare bias in codon usage in each genotype we also used the statistic ${\overset{―}{\Delta RSCU}}_{+}$ (Figure 3). Although no difference was detected in *F*_op_ among genotypes, there was a significant effect of mating system on ${\overset{―}{\Delta RSCU}}_{+}$ (*F*_1,94_ = 5.78, *P* < 0.05). The mean for all three selfers combined $\left({{\overset{―}{\Delta RSCU}}_{+}}_{\;\text{Selfers}}=0.24\right)$ was significantly lower than for the Brazilian outcrosser $\left({{\overset{―}{\Delta RSCU}}_{+}}_{\text{Brazil}}=0.29\right)$ (*t* = 1.99, *P* < 0.01). This pattern appears to have been largely driven by the significant effect of genotype (*F*_3,92_ = 3.02, *P* < 0.05) due to the lower ${{\overset{―}{\Delta RSCU}}_{+}}_{\;\text{Nicaragua}}\left(0.22\right)$ relative to the corresponding value of ${{\overset{―}{\Delta RSCU}}_{+}}_{\text{Brazil}}=0.29$ from Brazil (Figure 3; Tukey-Kramer HSD q* = 2.62, *P* < 0.01).

### Preferred to unpreferred synonymous changes

**Figure 4:**
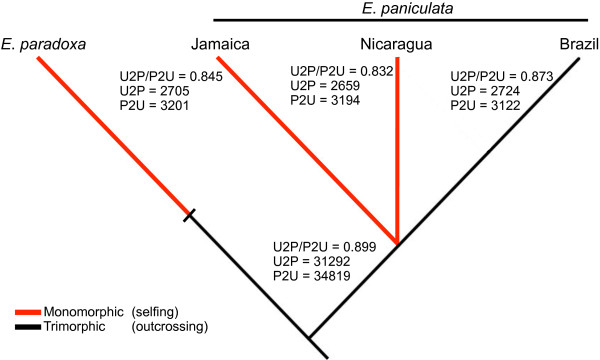
**Counts and ratio of preferred (U2P) and unpreferred (P2U) synonymous mutations in selfing and outcrossing genotypes of *Eichhornia, *as well as the ancestral branch connecting *E*. *paniculata *to *E*. *paradoxa.*** The ancestral state is considered to be the nucleotide observed at each position in *E. paradoxa*. The colours along the branches reflect the putative model of change in mating system although the exact timing of the transition along each branch is not known. The branch lengths are not to scale.

We compared the patterns of mutation between preferred and unpreferred codons in each genotype as an additional test for changes in selection on codon usage (Figure 4). There was a slight reduction in the proportion of preferred to unpreferred changes in synonymous codon use in the selfing genotypes relative to the root branch of *E*. *paniculata*. Both selfers, Jamaica (U2P/P2U = 0.845, *χ*^*2*^ = 5.04, 2 DF, *P* < 0.05) and Nicaragua (U2P/P2U = 0.832, *χ*^*2*^ = 7.74, 1 DF, *P* < 0.01), had a significantly lower proportion of preferred synonymous changes when compared to the ancestral lineage leading to *E. paniculata* (U2P/P2U = 0.899). The ratio of preferred to unpreferred changes in the outcrossing genotype from Brazil was not significantly different from the branch connecting *E*. *paniculata* to *E. paradoxa* (U2P/P2U = 0.873, *χ*^*2*^ = 1.14, 1 DF, *P* = 0.286). However, the ratio U2P/P2U of the outcrossing genotype was intermediate to the selfers and the ancestral lineage and was not significantly different from either selfer.

## Discussion

Large numbers of loci are required to detect significant shifts in patterns of selection across the genome accompanying the transition from outcrossing to selfing. This is largely because the mutational process is inherently noisy and many selfing populations are recently derived. As a result, most studies have failed to detect a significant signature of the effects of a transition to selfing on the efficacy of natural selection [20-25]. Here, we sequenced ~8000 genes from three independently derived selfing lineages and one outcrosser from *Eichhornia*, a plant genus that illustrates multiple independent evolutionary transitions from outcrossing to selfing. Below we discuss the implications of our results in the context of selective constraints on protein sequences and the underlying codon usage during mating system transitions.

Our study was based on a small number of genotypes, in part because of the large number of genes that we were interested in sampling. As a result, accurate estimates of polymorphism were not possible and this restricted the types of analyses that we could conduct. However, it is important to appreciate that our sampling scheme involving many loci provides a large sample of the deeper coalescent history of each population because, by definition the coalescent histories of unlinked loci are independent. Therefore, the specific identity of individuals sampled in each population is not important. In the case of the outcrossing genotype, this was sampled from a region of Brazil dominated by outcrossing populations with little evidence of population structure and higher levels of gene flow than are evident elsewhere in the range, where selfing populations occur more commonly [10,54]. As a result, it seems reasonable to assume, given the absence of any obvious population subdivision in the region from which this individual was sampled, that the loci sequenced provide a reasonable approximation of populations in this region, and thus are representative of outcrossing genotypes in general.

### Mating system and protein evolution

When analysing the number of non-synonymous mutations we found a trend for a higher fraction of deleterious mutations in the selfing genotypes of *E. paniculata* (Figure 1). When we considered all polymorphic non-synonymous sites, the two selfing lineages had a significantly higher fraction of sites considered deleterious. This result is consistent with the prediction that selfers are less efficient at purging deleterious mutations from populations. The trend was strongest in the Nicaraguan genotype, which also had a significantly higher proportion of non-synonymous to synonymous changes in comparison with the outcrossing genotype from Brazil. Interpretation of these values should take into account that not all changes observed are necessarily fixed within the respective populations from which the genotypes were sampled. In fact when we excluded heterozygous sites, which by definition are polymorphic, we found a significant elevation in the proportion of non-synonymous to synonymous polymorphisms in both selfing genotypes (the *P*_*N*_/*P*_*S*_ ratio rises from 0.41 in the outcrosser to 0.43 in the Jamaican selfer and 0.47 in the Nicaraguan selfer, χ^2^ = 18.3, 2 DF, *P* <0.001). Because the outcrosser had more heterozygous sites, the removal of these variants is likely to have preferentially removed slightly deleterious polymorphisms from this sample. A recent study of polymorphism in the derived selfer, *Capsella rubella*, detected an excess of rare non-synonymous mutations (SI Wright, unpublished data) and similarly an elevation in potentially deleterious polymorphisms was found in small, marginal population of *A. thaliana*[55]. If a similar pattern occurs in the Jamaican and Nicaraguan genotypes, some portion of the observed non-synonymous mutations could be due to polymorphism. However, our data are consistent with these mutations segregating for a longer time or at higher frequencies in the selfing populations, potentially due to a reduction in the efficiency of selection in purging deleterious mutations. Future sampling of more individuals from selfing and outcrossing populations of *E. paniculata* should allow an evaluation of the generality of our findings.

### Mating system and selection for codon bias

Differentiation of codon usage between high and low expression genes provided strong evidence of codon bias among our four samples. The degree of bias was partially explained by genotype and mating system (Figure 3). The measure ${\overset{―}{\Delta RSCU}}_{+}$[23] provides a useful way to compare codon bias across species or genotypes and compare the differentiation in codon usage between high and low expression genes. The outcrossing *E*. *paniculata* genotype from Brazil had a significantly higher ${\overset{―}{\Delta RSCU}}_{+}$ estimate than the three selfing genotypes combined. Moreover, while there was no significant difference between the outcrosser and the selfing genotype from Jamaica and *E*. *paradoxa*, there was a significant difference between the outcrosser and the Nicaraguan genotype. This pattern is illustrated in Figure 2, in which the Nicaraguan genotype appears less polarized in *ΔRSCU* values between optimal and sub-optimal codons. It is possible that a different amount of time has elapsed since the origin of selfing in these lineages, or the effective size of the Nicaraguan population may be much smaller than the Jamaican population. We found a similar trend in the analysis of protein coding changes where only the Nicaraguan genotype had a significantly higher ratio of non-synonymous to synonymous changes, and also had the highest fraction of deleterious mutations. Interestingly, the Nicaraguan genotype has fewer SNP differences in comparison with the outcrosser than the Jamaican genotype. This is consistent with a more recent common ancestor between these lineages and could indicate that selfing evolved more recently in this lineage. However, the time since the lineages diverged is not necessarily coincident with the evolution of selfing and we cannot exclude the possibility that the Nicaraguan genotype has been selfing longer or at a higher rate than the genotype from Jamaica. Consistent with this suggestion, the Nicaraguan genotype had less than half the proportion of heterozygous sites relative to the Jamaican genotype. The Nicaraguan genotype originated from one of only four populations of this species reported from Central America, unlike Jamaica where *E. paniculata* is more widely distributed and both selfing and outcrossing variants occur [38,56]. Therefore, it is possible that the underlying distribution of mutational effects on fitness is such that the reduction in *N*_e_ in the Jamaican population was not sufficient to render deleterious mutations effectively neutral.

We analyzed the fraction of synonymous mutations in each genotype that resulted in either changes to preferred or unpreferred codons. The patterns we obtained are in accord with our other results and suggest a small change in the efficacy of selection on codon usage in the two selfing genotypes of *E. paniculata*. Both had a significantly lower ratio of preferred over unpreferred changes compared to the ancestral branch, and there was no significant decline in the outcrossing genotype (Figure 4). If codon bias is at equilibrium with respect to mutation and selection, the number of preferred and unpreferred substitutions is expected to be equal [31]. However, this ratio in our samples, including the root branch, was below 1.0 (0.832-0.899). This may be explained by the contribution of mildly deleterious mutations segregating in each population. Consistent with this interpretation is the finding that the outcrossing lineage from Brazil had more unpreferred changes than the ancestral branch. The ratio of preferred to unpreferred fixations along the ancestral branch was also lower than 1.0, which may reflect a relaxation of selection in the selfing *E*. *paradoxa*. We cannot quantify this effect without another outgroup sequence because when the transition to selfing occurred along this branch in *E*. *paradoxa* is unknown. However, even if segregating mutations are contributing to the pattern, the fact that they are contributing more in selfing populations implies that mildly deleterious mutations are at higher frequencies, a pattern predicted by a relaxation of selection in selfing populations.

### Factors influencing the efficacy of selection in selfers

One explanation for the stronger effect of selfing on codon bias relative to protein evolution is that the underlying distributions of fitness effects (DFE) of synonymous and non-synonymous mutations are likely to be different. Most models predict an accumulation of deleterious mutations in inbreeders and rely on a large class of slightly deleterious mutations with additive fitness effects [28,57]. However, if a large fraction of non-synonymous mutations are strongly deleterious such that *N*_*e*_*s* remains greater than 1.0, the rate of fixation may not be elevated. Therefore, if the shape of the DFE for synonymous sites differs, so that a large portion of the mutations altering codon usage are very weakly selected, then only a small decline in *N*_*e*_ due to selfing would cause *N*_*e*_*s* < 1. Weak selection on codon bias seems likely given the relatively low estimates of *N*_*e*_*s* in other species using the same method (*e.g. Drosophila* spp. *N*_*e*_*s* = −0.37 – 1.74 [58]; *Populus* spp, *N*_*e*_*s* = −0.232 – 0.720 [59]). These results are what would be expected with the relatively weak effect of selfing we found for the accumulation of non-synonymous mutations and the small reductions in selection for codon bias evident in our data.

Models that predict an accumulation of deleterious mutations in selfing populations generally require that mutations are additive in their effects [20]. However, if a sizable fraction of slightly deleterious mutations are recessive they will be more effectively eliminated in highly selfing populations through purging [60,61]. Barrett and Charlesworth [62] reported differences in the intensity of inbreeding depression between selfing Jamaican and outcrossing Brazilian populations of *E. paniculata* after five generations of controlled selfing and outcrossing. They interpreted their results as being consistent with the occurrence of natural purging of genetic load in the Jamaican population owing to a history of selfing. We may therefore expect that some mutations identified in our outcrossing genotype are polymorphic and that sheltered genetic load may have been purged during the colonization bottleneck associated with long-distance dispersal to the Caribbean by *E*. *paniculata.* If true, this could lessen the signal of deleterious mutation accumulation in selfers and potentially explain the weaker signal found in the Jamaican genotype.

Our results suggest that the accumulation and/or segregation of deleterious mutations is different between the two selfing genotypes of *E. paniculata*. This may be because selfing rates vary considerably within and among Jamaican populations (*e.g.*[39]) and even low levels of outcrossing are sufficient to allow recombination to lessen many of the predicted consequences of extreme inbreeding (*e.g.*[63,64]). We have no estimate of the selfing rate or amount of nucleotide diversity in the Nicaraguan population from which our sample originated. Flowers produced by plants from this population are the smallest known in *E*. *paniculata* and are very likely to be highly autaogamous given their strong facility for self pollination in the glasshouse. It is possible that the significantly reduced value of ${\overset{―}{\Delta RSCU}}_{+}$ compared to the outcrosser is a result of a more severe reduction of *N*_*e*_ in this population than in the Jamaican population, as discussed earlier. A clearer signal for the accumulation of deleterious mutations in *E*. *paniculata* may not exist if the transition from outcrossing to selfing occurred recently. It is estimated that the colonization of the Caribbean from Brazil occurred ~125,000 generations ago [10], perhaps leaving insufficient time to reach equilibrium and for ancestral polymorphism to coalesce within selfing populations. Our findings indicate a higher fraction of deleterious mutations in selfing lineages of *E*. *paniculata*. The effects of these mutations on fitness and how many must accumulate to impact the viability of selfing lineages is unknown.

## Conclusions

Here, we use high-throughput sequencing of the floral transcriptome of the aquatic plant *Eichhornia paniculata*, and its sister species *E. paradoxa*, to investigate whether a transition to self-fertilization is associated with a reduction in the efficacy of natural selection. We observed a small increase in the proportion of non-synonymous mutations in one of the selfing *E. paniculata* genotypes. However, in both *E. paniculata* selfing genotypes the proportion of non-synonymous changes inferred to be deleterious was significantly higher than in the outcrossing genotype. A key result from our study is the finding of a reduction in codon bias usage in selfing genotypes, as predicted by a relaxation in selection efficacy. Our results are therefore consistent with theoretical expectations, and highlight the value of high-throughput data to investigate long-standing questions in evolutionary genetics.

## Competing interests

The authors declare that they have no competing interests.

## Authors’ contributions

RWN and MS were responsible for the sequence assembly, alignment and annotation. RWN was primarily responsible for the analysis of the codon bias and MS for the analysis of protein coding changes. All authors contributed to the preparation of the manuscript and have read and approved the final manuscript.

## Supplementary Material

Additional file 1 SNP validation and error rate estimation. Click here for file
