# Supplementary material for: Genomic consequences of transitions from cross- to self-fertilization on the efficacy of selection in three independently derived selfing plants
Source: BMC Genomics. 2012 Nov 12;13:611. doi: 10.1186/1471-2164-13-611 (PMC3533693; doi:10.1186/1471-2164-13-611)
Supplement: Additional file 1 — SNP validation and error rate estimation. [file 1471-2164-13-611-S1.docx]

#### **Supplementary Material**

#### **SNP validation and error rate estimation**

To estimate the accuracy of our SNP calls we compared the genotypes of each of the three *E*. *paniculata* strains inferred from Illumina data to orthologous Sanger sequenced loci. The Sanger sequence data were generated by PCR amplifying loci from nuclear DNA, which contained a mixture of coding and intronic sequence. The resulting amplicons were then analyzed using standard Sanger sequencing (for details of Sanger sequences see Ness et al. 2010). In total there were seven ‘Sanger’ loci that had orthologous ESTs in our ‘Illumina’ transcriptome sequences. We aligned the coding regions of each of the Sanger loci to their orthologous Illumina ESTs using Muscle (Edgar 2004) and verified the alignments manually. For each of the three genotypes we examined all base calls that did not agree between the two sequencing platforms to assess the source of each discrepancy and the overall error rate.

Across the seven Sanger sequences we aligned 2659 bp of coding sequence in each of the three genotypes for a total of 7977 bp of comparable sequence. There were a total of three bases at two alignment positions that differed between the Sanger and Illumina genotype calls. The first two discrepancies were at the same position in the Jamaican and Brazilian genotypes. The Illumina genotypes were called by aligning paired end reads to consensus contigs generated by *de novo* assembly (see Methods). However, the contig generated by our *de novo* assembly had the same genotype at the position in question as the Nicaraguan sample. Therefore, it appears that although the Nicaraguan genotype was called correctly, read mapping of the Brazilian and Jamaican samples did not agree with the Sanger sequence at this variant site. The second position was in the Brazilian genotype where the Sanger sequence was a heterozygote C/T and the Illumina was a homozygote T. Upon closer examination 4 of 23 Illumina reads carried the C allele, but the algorithm used in Maq/SamTools called this a homozygote. Sampling effects can make accurately distinguishing a true heterozygote from read mapping or sequencing errors difficult (Keightley and Halligan 2011). Therefore, it is likely that this represents a sampling error in which one of two alleles at this site was under-represented leading to an erroneous genotype call. However, polymorphism in priming sites can lead to similar problems when using Sanger sequencing and it is not clear that Sanger sequencing is more reliable when calling heterozygous positions. We therefore had one alignment position which was a false negative SNP (Brazil and Jamaica erroneously marked as the same allele as Nicaragua), and another false negative where the Illumina data missed one of the two alleles at a heterozygous site. There are a number of ways of expressing the error rate but with the limited data available we estimate an error rate of 3 in 7977 (3.8x 10^-4^/site). Alternatively, this could be expressed as two false negatives in 2659 alignment positions. This will introduce errors at a rate substantially lower than either polymorphism or divergence in our samples and is therefore unlikely to significantly alter any inferences made in this study.

Another method to determine the effect of error on the analyses is to vary the quality thresholds used when identifying SNPs in the sequences. To test whether this has an effect we used genetic diversity (*θ*_π_) as a metric and observed how it changed with quality. We did this by generating the full transcriptome for each individual under a range of ‘Genotype Quality’ (GQ) cutoffs (GQ > 10, GQ > 20, GQ > 30). The GQ is the Phred-scaled confidence that the genotype call is correct. We found that the level of diversity declines with increasing stringency (*θ*_GQ>30_ = 0.0074, *θ*_GQ>20_ = 0.0076, *θ*_GQ>10_ = 0.0078). However, differences at contrasting quality thresholds may not be entirely due to error. The GQ score is strongly influenced by the depth of coverage, and coverage is a function of gene expression. Because highly expressed genes tend to be more selectively constrained by purifying selection on protein sequence and codon usage we conducted an ANOVA to determine what fraction of the variance in diversity is attributable to the quality cutoff after controlling for expression by treating it as a covariate in the model. No significant expression by treatment interaction was detected and it was therefore excluded from the model. This revealed that only 0.6% (*F*_2,1_ = 5.4891, *P* >  0.01) of the variance in diversity was attributable to the quality threshold imposed, and indicates that although the quality cutoff does affect polymorphism it only explains a very small fraction of the overall variance. Moreover, while the decline in diversity at higher GQ cutoffs could be interpreted as a reduction in the false positive rate, there is another potentially more important factor downwardly biasing estimates of diversity under very stringent quality thresholds. Specifically, like most SNP calling algorithms GQ is only reported at potentially variable sites. Therefore, excluding some of the 'potentially' variant sites will disproportionately affect variant rather than invariant sites and reduce diversity due to false negatives. It is therefore important not to uncritically apply higher quality thresholds in studies of this type because it could create a strong bias towards highly expressed genes and thus erroneously exclude variant sites.

References

Edgar RC. 2004. MUSCLE: multiple sequence alignment with high accuracy and high throughput. Nucleic Acids Research 32:1792-1797.

Keightley PD, and Halligan DL. 2011. Inference of site frequency spectra from high-throughput sequence data: quantification of selection on nonsynonymous and synonymous sites in humans. Genetics 188:931-940.

Ness, RW, Wright SI, and Barrett SCH. 2010. Mating-system variation, demographic history and patterns of nucleotide diversity in the tristylous plant *Eichhornia* *paniculata*. Genetics 184:381-392.
